# Transplantion of Mucous Membrane

**Published:** 1871-12

**Authors:** 


TRANSPLANTION OF MUCOUS MEMBRANE:—At a meeting of the College of Physicians and Surgeons of Vienna, Dr. Czerny (*Am. Practioner*) exhibited a case of transplantation of the mucous membrane of the nose upon a granulating surface of the upper arm. He spoke of three cases he had experimented on in this way, and one in which he had taken the mucous membrane from the mouth. The mucous membrane in one instance was taken from an extirpated polypus, and transplated two hours after its removal. It had lost on its new ground its villi, and had changed into basement epithelium.—*N. Y. Medical Journal.*

